# Cytoreductive nephrectomy for metastatic renal cell carcinoma: inequities in access exist despite improved survival

**DOI:** 10.1002/cam4.1137

**Published:** 2017-08-22

**Authors:** Manish I. Patel, Kieran Beattie, Albert Bang, Howard Gurney, David P. Smith

**Affiliations:** ^1^ Discipline of Surgery Westmead Hospital University of Sydney Sydney Australia; ^2^ Department of Urology Westmead Hospital Westmead Australia; ^3^ Cancer Research Division Cancer Council NSW Sydney Australia; ^4^ Faculty of Medicine and Health Sciences Macquarie University Sydney Australia; ^5^ Sydney Medical School The University of Sydney Sydney Australia; ^6^ Menzies Health Institute Queensland Griffith University Nathan Australia

**Keywords:** Cytoreductive, inequity, laparoscopic, metastatic, Renal cell carcinoma, survival

## Abstract

The use of cytoreductive nephrectomy (CRN) in the targeted therapy era is still debated. We aimed to determine factors associated with reduced use of CRN and determine the effect of CRN on overall survival in patients with metastatic renal cell carcinoma (RCC). All advanced RCC diagnosed between 2001 and 2009 in New South Wales, Australia, were identified from the Central Cancer Registry. Records of treatment and death were electronically linked. Follow‐up was to the end of 2011. Multivariable logistic regression analysis was used to determine factors associated with the receipt of CRN. Cox proportional hazards model was used to determine factors associated with survival. A total of 1062 patients were identified with metastatic RCC of whom 289 (27%) received CRN. There was no difference in the use of CRN over the time period of the study. Females (OR 0.68 (95% CI: 0.48–0.96)), unmarried individuals (OR 0.68 (95% CI: 0.48–0.96)), treatment in a nonteaching hospital (OR 0.26 (95% CI: 0.18–0.36)) and individuals without private insurance (OR 0.29 (95% CI: 0.20–0.41)) all had reduced likelihood of receiving CRN. On multivariable analysis, not receiving CRN resulted in a 90% increase in death (HR 1.90 (95% CI: 1.61–2.25)). In addition, increasing age (*P* < 0.001), increasing Charlson comorbidity status (*P* = 0.002) and female gender also had a significant independent association with death. Despite a strong association with improved survival, individuals who are elderly, female, have treatment in a nonteaching facility or have no private insurance have a reduced likelihood of receiving CRN.

## Introduction

Historically patients presenting with metastatic renal cell carcinoma (mRCC) have a poor prognosis, with a 2 year survival of 10–20% 1. The role of cytoreductive nephrectomy (CRN) in the era of immunotherapies was well established being based on two randomized trials which demonstrated a survival advantage in selected patients who had mRCC and CRN 2, 3. Since the advent of targeted therapies in 2005 4 and a change in the first line therapy for mRCC from immunotherapy to tyrosine kinase inhibitors (TKI), the role of CRN has been questioned 5.

A study from the National Cancer Data Base (NCDB) has demonstrated that the rate of CRN increased to a maximum of 48% in 2005 but rapidly started decreasing following the widespread usage of TKI systemic therapies 6.

Randomized trials investigating the role of CRN in mRCC are ongoing and have not yet reported, but evidence is accumulating that there is still an ongoing role for CRN in the management of mRCC 7. We sought to investigate the trends in use of CRN on a population basis in Australia and identify factors that might influence its use.

## Methods

### Patients

Data for all cases of kidney cancer diagnosed in New South Wales (NSW) were obtained from the NSW Central Cancer Registry (CCR). Operational details of the Registry have been described previously 8. Notifications to the CCR of kidney cancer (ICD‐10 code: C64) diagnosed in NSW are mandated from pathology laboratories, hospitals and other treatment centers under the NSW Public Health Act 1991. All patients had histological confirmation of RCC. For this study all registered cases diagnosed between January 2001 and December 2009 were eligible. Hospital admission and treatment details were retrieved by linkage to the NSW Admitted Patient Data Collection (APDC) on all hospital separations in NSW in the period January 2001 to end of December 2011. Hospital medical coders abstracted individual patient information from medical records following the patient's discharge from any hospital in the state of NSW. This includes dates of admission and separation, procedures undertaken and diagnoses relating to the hospital episode. Death information was obtained by electronic linkage of the records from the CCR with NSW death records from the Registry of Births Deaths and Marriages. (January 2001 to end of December 2011) and Australia Coordinating Registry Cause of Death Unit Record File (January 2001 to end of October 2007). Linkage of records in these data sets was carried out by the Centre for Health Record Linkage (CHeReL), using probabilistic matching carried out with ChoiceMaker software (ChoiceMaker Technologies Inc., New York, US). This linkage was performed using name, address, date of birth, date of diagnosis and hospital‐recorded clinical information that identified cases common to all data sets, and clerical reviews for questionable matches were undertaken by trained staff within the CHeReL.

Only individuals diagnosed with metastatic (distant) disease were included in this analysis. Distant stage at diagnosis was based on the CCR classification of stage at first presentation. This was determined as the maximum extent of the cancer based on all reports and notifications undertaken within four months from diagnosis. This classification follows the international coding guidelines for summary stage adopted by the World Health Organization and the International Association of Cancer Registries 9.

Individuals were classified by: (1) age at diagnosis categorized in decades; (2) gender; (3) marital status‐ married (including de‐facto) verses other; (4) Modified Charlson comorbidity score. An additional score was not allocated for metastatic disease; (5) socio‐economic status (SES), three ordinal categories using the residential local government area‐based Socio‐economic Index of Relative Disadvantage for Areas (SEIFA) 10; (6) two ordinal categories using the residential local government area‐based Accessibility/Remoteness Index of Australia (ARIA) 11; (7) treating hospital type (public verses private) and treating hospital type (teaching hospital (had a training urologist) versus nonteaching).

### Statistical analysis

Differences were measured using chi‐squared analysis. Multivarivable logistic regression analysis was used to analyze predictors of receiving CRN. Multivariable survival analyses were performed with the Cox proportional hazard regression model. Statistical significance in this study was considered at *P* < 0.05. All reported *P* values are two‐sided.

All analyses were carried out in SAS version 9.3 (SAS Institute Inc., Cary, NC, US). The NSW Population and Health Services Research Ethics Committee approved this study (Approval number HREC/10/CIPHS/21).

### Ethical Standards

All procedures performed in studies involving human participants were in accordance with the ethical standards of the institutional and/or national research committee and with the 1964 Helsinki declaration and its later amendments or comparable ethical standards.

## Results

Between the years of 2001 and 2009, 1062 individuals were diagnosed with metastatic kidney cancer in the state of NSW. The median follow‐up time for this study was 5 months, and a median of 52 months for those still alive. At the end of follow‐up, 94 (9%) were alive, 643 (61%) had died from kidney cancer, 110 (10%) were dead from other causes and 215 (20%) were dead from unknown causes.

**Figure 1:**
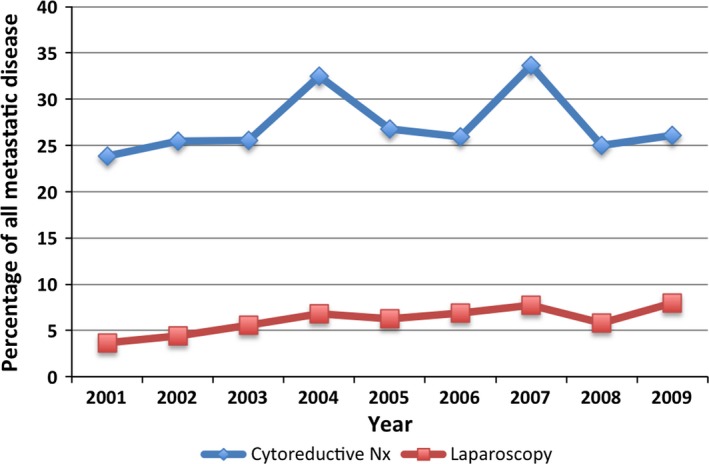
Prevalence of cytoreductive nephrectomy and laparoscopic cytoreductive nephrectomy by year of diagnosis.

In total 289 (27%) received CRN. Figure 1 demonstrates the proportion of patients with metastatic kidney cancer that received CRN by year and also the proportion that had it performed by laparoscopy. Between the years 2001 and 2009 there was no overall change in the proportion receiving CRN, however, there was a slight increase in laparoscopic CRN from 2001 (3.7%) to 2009 (8.0%) (*P* = 0.15).

The patient characteristics of those who received, and those who did not receive CRN are given in Table 1. Cytoreductive nephrectomy was more common in those of younger age (*P* < 0.001). There was also a higher propensity for males to receive CRN compared to females (*P* < 0.001). Married individuals were also more likely to receive CRN as were those with less comorbidities (*P* < 0.001). There was no difference in CRN rates between different socio‐economic groups, place of residence or hospital type (public vs. private). Teaching hospitals however performed a higher proportion of CRN compared to nonteaching hospitals (*P* < 0.001).

**Table 1:** Patient characteristics

	Cytoreductive nephrectomy (%)	No nephrectomy (%)	Total	*P*‐value
Number	289 (27.2)	773 (32.8)	1062	
Age group				<0.001
<50	62 (21.5)	39 (5.0)	101	
50–59	75 (26.0)	110 (14.2)	185	
60–69	77 (26.6)	165 (21.3)	242	
70–79	60 (20.8)	216 (27.9)	276	
80+	15 (5.2)	243 (31.4)	258	
Gender				<0.001
Male	221 (76.5)	471 (60.9)	692	
Female	68 (23.5)	302 (39.1)	370	
Marital status				<0.001
Married	208 (72.0)	425 (55.0)	633	
Other	81 (28.0)	348 (45.0)	429	
Charlson Comorbidity (modified)				<0.001
0	185 (64.0)	393 (50.8)	578	
1	36 (22.5)	133 (17.2)	169	
2+	68 (23.5)	247 (32.0)	315	
Socio‐economic status (SEIFA)				0.076
1 (High)	94 (32.5)	198 (25.6)	292	
2 (Middle)	92 (31.8)	263 (34.0)	355	
3 (Low)	103 (35.6)	312 (40.4)	415	
Place of residence				0.202
Major cities	199 (68.9)	500 (64.7)	699	
Other	90 (31.1)	273 (35.3)	363	
Hospital type				0.053
Public	191 (66.1)	558 (72.2)	749	
Private	98 (33.9)	215 (27.8)	313	
Hospital type				<0.001
Teaching	149 (51.6)	230 (29.8)	379	
Nonteaching	140 (48.4)	543 (70.2)	683	
Status				
Alive	58 (20.1)	36 (4.7)	94	
Died of RCC	147 (50.9)	496 (64.2)	643	
Died other causes	21 (7.3)	42 (5.8)	110	
Unknown	63 (21.8)	152 (24.1)	215	

Logistic regression analysis of potential factors associated with the use of CRN are given in Table 2. Age was a significant independent factor associated with whether CRN was undertaken (*P* < 0.001). Compared to individuals aged <50 years, those aged 50–59 years had a 65% reduced likelihood of receiving CRN (Odds Ratio (OR) 0.35 (95% Confidence Interval (CI): 0.20–0.60)) and for those aged 80 years or over, the likelihood was reduced 96% (OR 0.04 (95% CI: 0.02–0.07)).

**Table 2:** Multivariable model of predictors of receiving cytoreductive nephrectomy

Factor	OR (95% CI)	*P*‐value
Age		<0.001
<50	1	
50–59	0.35 (0.20–0.60)	
60–69	0.26 (0.16–0.45)	
70–79	0.17 (0.10–0.29)	
80+	0.04 (0.02–0.07)	
Gender		0.029
Male	1	
Female	0.68 (0.48–0.96)	
Marital Status		0.027
Married	1	
Other	0.68 (0.48–0.96)	
Charlson Comorbidity		0.265
0	1	
1	0.71 (0.45–1.13)	
2+	0.82 (0.57–1.18)	
Hospital type		<0.001
Teaching	1	
Nonteaching	0.26 (0.18–0.36)	
Insurance status		<0.001
Private	1	
Public	0.29 (0.20–0.41)	

Females also had a 32% lower likelihood of receiving CRN (OR 0.68 (95% CI: 0.48–0.96)) compared to males. Unmarried individuals had a similar risk to females (OR 0.68 (95% CI: 0.48–0.96)). Having treatment in a nonteaching hospital also reduced the likelihood of receiving CRN by 74% (OR 0.26 (95% CI: 0.18–0.36)). Individuals without private insurance had a 71% reduced likelihood of receiving a CRN (OR 0.29 (95% CI: 0.20–0.41)). Charlson comorbidity did not have a significant effect on receiving CRN (*P* = 0.265).

Multivariable analysis on overall survival was performed using the Cox proportional hazards model (Table 3. Increasing age (*P* < 0.001) and increasing Charlson comorbidity status (*P* = 0.002) had a significant influence on death. Female gender increased the risk of death by 19% (Hazard Ratio (HR): 1.19 (95% CI: 1.03–1.36)). Being unmarried (*P* = 0.656) and having treatment in a nonteaching hospital (*P* = 0.471) did not affect the risk of death. Importantly, after adjusting for the influence of age, comorbidity, gender, hospital type and marital status, not having a CRN increased the risk of death by 90% (HR: 1.90 (95% CI: 1.61–2.25)). We further sought to determine whether the period of treatment (pre targeted era vs. post targeted era) had any effect on survival in those that received and did not receive CRN, however no difference was identified between any of the groups (*P* = 0.26).

**Table 3:** Multivariable model predicting death in patients with metastatic kidney cancer

Factor	HR (95%CI)	*P*‐value
Age		<0.001
<50	1	
50–59	1.01 (0.77–1.33)	
60–69	1.07 (0.83–1.4)	
70–79	1.29 (0.99–1.67)	
80+	1.65 (1.26–2.16)	
Gender		0.016
Male	1	
Female	1.19 (1.03–1.36)	
Marital status		0.656
Married	1	
Other	1.03 (0.9–1.18)	
Charlson Comorbidity (modified)		0.002
0	1	
1	1.12 (0.93–1.35)	
2+	1.31 (1.13–1.52)	
Hospital type		0.4711
Teaching	1	
Nonteaching	1.05 (0.92–1.21)	
Cytoreductive nephrectomy		<0.001
Yes	1	
No	1.90 (1.61–2.25)	

## Discussion

In this large population based series of 1042 patients with mRCC, only 27% received CRN; without any change in the rate of CRN over the years 2001 to 2009. The rate of laparoscopic CRN, however, has increased from 3.6% in 2001 to 7.9% in 2009.

Our findings are quite different to those reported in the SEER database in the immunotherapeutic era, where CRN rates were increasing each year until study end in 2004, where 40% of mRCC patients were receiving CRN 12. This was also supported by a study from the NCDB which ranged from 2000 to 2008 6. In this study, rates of CRN increased every year by 3% until 2005 when systemic treatment paradigm changed and subsequently decreased by 3% a year to 2008. Targeted therapies have been utilized in Australia since 2005. Is not clear why the rate of CRN usage in Australia is substantially lower than the USA, or why the trends identified before and after 2005 have not been observed in Australia. One potential explanation is that in the United States prior to 2005 IL‐2 was frequently administered in addition to CRN. In Australia IL‐2 was not available and treatment with Interferon was the standard and CRN was not routinely administered with this therapy.

It was however pleasing to see that the minimally invasive approach to CRN surgery more than doubled to 31% of all CRN in 2009 and reflects a general increase in the use of minimally invasive kidney surgery in the state over this time period 13. This rate is lower than 41% rate of CRN reported in a BAUS study of CRN so there is certainly room for improvement 14. The surgery of mRCC is technically more challenging than localized disease as they often have invasion of lymph nodes, major vessels and adjacent organs. This explains why the rate of laparoscopy was 31% compared to 60% for localized disease in a study we have previously reported 13. These operations can be performed with effective open exposure using mini‐flank or mini sub costal approaches 15. In the end CRN should be performed whenever possible by whatever technique the surgical team is most comfortable with.

Our study identified a number of factors associated with the use of CRN. An inverse association with increasing age is an expected finding, with patients in their 70s and 80s experiencing a 83% and 96% reduction in rates of CRN compared to those less than 50 years. Similar findings were identified in the NCDB database 6 and was presumed to be due to increasing comorbidity status. Our study ,however, does not support that premise as increasing age was independent of comorbidity and infact comorbidity was not a significant factor determining use of CRN (*P* = 0.265).

A surprising factor associated with a 32% reduced likelihood of receiving CRN was female gender. This finding was also observed in the SEER database study 12 and is not clear why. In a separate study, we have identified that females with localized RCC have a lower likelihood of receiving partial nephrectomy as well and may reflect a lower likelihood of females receiving kidney surgery in general 13. Another surprising factor was that unmarried patients had a 32% reduced risk of receiving CRN. This has not been reported previously in this setting, but may reflect the observation in many cancers that married patients receive more treatment and better outcomes 16, 17.

Our study also identified that patients without private insurance had a 91% reduced likelihood of receiving CRN, a finding also identified in the NCDB 6. While the true reasons for this are not known, less specialized care and longer surgical waiting lists may be contributing factors. Patients treated outside teaching hospitals also experience a 74% lower likelihood of receiving CRN which again we hypothesize may be due to less specialized care and reduced willingness for surgeons to do complex surgery in lower volume hospitals, a finding we have also observed with partial nephrectomy and laparoscopy in localized disease 13.

Multivariable analysis also revealed that lack of CRN was an independent risk factor for death, after adjusting for age, gender, comorbidity, marital status and hospital type. The risk of death increased by 90% in those that did not receive a CRN. The benefit of CRN in the targeted therapy era has been controversial. It is important to note that the clinical trials that led to the approval of seven targeted therapies currently available had prior nephrectomy in the vast majority of patients 4, 18, 19, 20, 21, 22 and hence the benefit of these agents must be placed in the context of prior CRN. A study by Choueiri has shown a significant overall survival advantage for mRCC patients treated with targeted agents undergoing CRN if they had favorable or intermediate prognostic features 23. The true benefit of CRN in the targeted therapy era will be answered by the two randomized trials which have yet to report, CARMENA (NCT00930033) and SURTIME (NCT01099423).

The present study's strength is that it is population based and collected every diagnosed mRCC in the New South Wales, Australia during the study period. The results are generalizable to the whole State, and possibly the country, as they reflect not just academic centers, where most outcomes studies originate but all locations where surgery is performed. In addition, every inpatient admission is recorded and matched through high‐quality data linkage techniques resulting in reliable levels of surgical treatment ascertainment. However, this study does have several limitations that may bias the results. The first, potential inaccuracies in the staging of patients, as the accuracy of staging information collected and recorded by the NSW Central Cancer Registry cannot be confirmed. The second is that detailed information on local cancer size and characteristics of metastatic disease are not collected by the Cancer Registry. Finally nonsurgical treatments to kidney tumors such as HIFU, Radio Frequency Ablation and embolization were not available for analysis. These characteristics may be important modifiers in the outcomes described in this paper.

In conclusion, this population based study demonstrates a low rate but unchanged trend in the use of CRN in Australia. Increasing age, female gender, unmarried status, having treatment in a nonteaching hospital and lack of private insurance decreased the likelihood of receiving CRN. Receipt of CRN independently improved overall survival.

## Conflict of Interest

This study was funded by Cancer Institute NSW (10/ECF/2‐29) and NHMRC (1016598). Author MIP has received honorarium from Ferring, Astra Zenaca, Astellas and Jannsen. Author HG has received honorarium from Astellas, Jannsen and Pfizer. No other conflict of interest exist.
